# (μ-Oxalato-κ^4^ *O* ^1^,*O* ^2^:*O* ^1′^,*O* ^2′^)bis­[bis­(2,2′-bipyridine-κ^2^ *N*,*N*′)cobalt(II)] μ_6_-oxido-dodeca-μ_2_-oxido-hexa­oxido-hexa­tungstate(VI)

**DOI:** 10.1107/S1600536810023007

**Published:** 2010-06-18

**Authors:** Congwen Shi, Liming Fan, Peihai Wei, Bin Li, Xiutang Zhang

**Affiliations:** a Advanced Material Institute of Research, Department of Chemistry, Qilu Normal University, Jinan 250013, People’s Republic of China; b College of Chemistry and Chemical Engineering, Liaocheng University, Liaocheng, 252059, People’s Republic of China

## Abstract

The asymmetric unit of the title compound, [Co_2_(C_2_O_4_)(C_10_H_8_N_2_)_4_][W_6_O_19_], consists of one half of the complex [Co_2_(C_2_O_4_)(C_10_H_8_N_2_)_4_]^2+^ cation and one half of the Lindqvist-type [W_6_O_19_]^2−^ isopolyanion. Both constituents are completed by crystallographic inversion symmetry. In the dimeric cation, the Co^II^ atom is surrounded in a distorted octa­hedral coordination by four N atoms from two chelating 2,2′-bipyridine ligands and by two O atoms from the chelating oxalate anion. The Lindqvist-type anion exhibits the characteristic W—O bond-length distribution, with the shortest bonds being the W—O_terminal_ bonds and the longest being those to the central O atom.

## Related literature

For general background to polyoxidometalates, see: Pope & Müller (1991 ▶). For polyoxidometalates modified with amines, see: Zhang, Dou *et al.* (2009 ▶); Zhang, Wei *et al.* (2009 ▶); Zhang *et al.* (2010 ▶). For another structure comprising a Lindqvist-type isopolyanion, see: Meng *et al.* (2006 ▶). For a related structure, see: Li & Xu (2009 ▶).
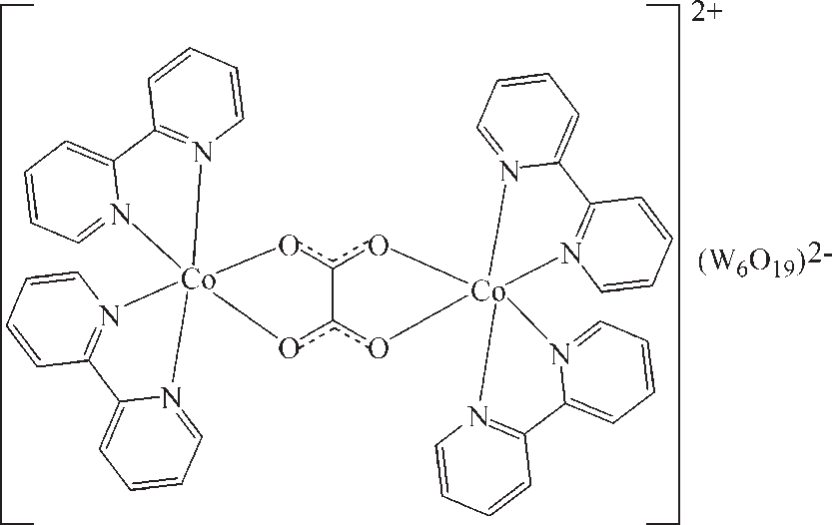


## Experimental

### 

#### Crystal data

[Co_2_(C_2_O_4_)(C_10_H_8_N_2_)_4_][W_6_O_19_]
*M* *_r_* = 2237.72
Triclinic, 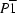

*a* = 9.4876 (15) Å
*b* = 9.8548 (15) Å
*c* = 14.174 (2) Å
α = 90.769 (2)°
β = 91.576 (2)°
γ = 91.113 (2)°
*V* = 1324.3 (4) Å^3^
*Z* = 1
Mo *K*α radiation
μ = 13.67 mm^−1^
*T* = 293 K
0.12 × 0.10 × 0.08 mm

#### Data collection

Bruker APEXII CCD diffractometer
Absorption correction: multi-scan (*SADABS*; Bruker, 2001 ▶) *T*_min_ = 0.291, *T*_max_ = 0.408
9331 measured reflections
4613 independent reflections
3755 reflections with *I* > 2σ(*I*)
*R*_int_ = 0.027

#### Refinement

*R*[*F* ^2^ > 2σ(*F* ^2^)] = 0.031
*wR*(*F* ^2^) = 0.076
*S* = 1.00
4613 reflections
368 parameters
H-atom parameters constrained
Δρ_max_ = 2.41 e Å^−3^
Δρ_min_ = −1.10 e Å^−3^

### 

Data collection: *APEX2* (Bruker, 2004 ▶); cell refinement: *SAINT-Plus* (Bruker, 2001 ▶); data reduction: *SAINT-Plus*; program(s) used to solve structure: *SHELXS97* (Sheldrick, 2008 ▶); program(s) used to refine structure: *SHELXL97* (Sheldrick, 2008 ▶); molecular graphics: *SHELXTL* (Sheldrick, 2008 ▶); software used to prepare material for publication: *SHELXTL*.

## Supplementary Material

Crystal structure: contains datablocks global, I. DOI: 10.1107/S1600536810023007/wm2358sup1.cif

Structure factors: contains datablocks I. DOI: 10.1107/S1600536810023007/wm2358Isup2.hkl

Additional supplementary materials:  crystallographic information; 3D view; checkCIF report

## Figures and Tables

**Table 1:** Selected bond lengths (Å)

Co1—O1	2.104 (6)
Co1—N1	2.101 (7)
Co1—N4	2.105 (6)
Co1—N2	2.114 (6)
Co1—N3	2.119 (7)
Co1—O2	2.134 (6)
O3—W2	1.690 (6)
O4—W2	1.919 (6)
O4—W3	1.926 (6)
O5—W3	1.904 (5)
O5—W2^i^	1.935 (5)
O6—W3	1.698 (6)
O7—W3	1.915 (6)
O7—W1	1.931 (6)
O8—W3^i^	2.3185 (4)
O8—W3	2.3185 (4)
O8—W1	2.3240 (4)
O8—W1^i^	2.3240 (4)
O8—W2^i^	2.3252 (5)
O8—W2	2.3252 (5)
O9—W2	1.912 (6)
O9—W1	1.915 (5)
O10—W1	1.914 (6)
O10—W3^i^	1.914 (6)
O11—W1	1.696 (6)
O12—W1	1.922 (5)
O12—W2^i^	1.920 (6)

Symmetry code: (i) 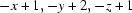.

**Fig. 1.**
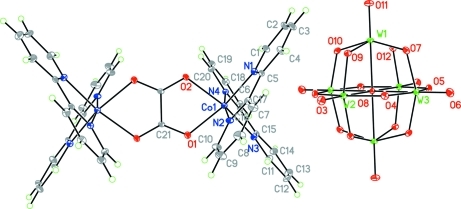
The cation and anion of the title compound with the atom-labelling scheme. Displacement ellipsoids are drawn at the 30% probability level; H atoms are given as spheres of arbitrary radius.

## supplementary crystallographic information

### Comment

There has been extensive interest in polyoxidometalates, owing to their fascinating properties and great potential applications in many fields such in catalysis, material science, medicine and magnetochemistry (Pope & Müller, 1991). Organic amines, such as 3-(2-pyridyl)pyrazole and pyrazine, are used to effectively modify polyoxidomolybdates or heteropolyoxidomolybdates under hydrothermal condictions (Zhang, Dou *et al.*, 2009; Zhang, Wei *et al.*, 2009; Zhang *et al.*, 2010). Here, we describe the synthesis and structural characterization of the title compound.

As shown in Figure 1, the title compound consists of two subunits, *viz.* of a binuclear complex [Co_2_(C_2_O_4_)(C_10_H_8_N_2_)_4_]^2+^ cation, and one Lindqvist-type [W_6_O_19_]^2-^ isopolyanion. Both constituents exhibit 1 symmetry. The Co^2+^ cation is surrounded in a distorted octahedral coordination by four N atoms from two chelating 2,2'-bipyridine ligands and two O atoms from a chelating oxalate anion. The Co—N and Co—O bond lengths are in the range of 2.101 (7)—2.119 (7) and 2.104 (6)—2.134 (6) Å, respectively, and are in good agreement with the bond lenghts observed for *catena*-poly[[(2,2'-bipyridine-*κ*N,*N*')cobalt(II)]-*µ*-oxalato- *κ*^4^O^1^,*O*^2^:*O*^1'^,*O*^2'^] (Li & Xu, 2009).

The [W_6_O_19_]^2-^ polyoxidoanion, possessing the well known Lindquist structure, is formed by six WO_6_ octahedra connected with each other through edge-sharing oxygen atoms. This anion approaches an approximate *O_h_* symmetry, but actually has 1 symmetry. Three different kinds of oxygen atoms exist in the cluster, *viz.* terminal Oa, double-bridging Ob, and central Oc oxygen atoms. Therefore, W—O bond lengths can be grouped into three sets: W—O*a*: 1.690 (6)—1.698 (6) Å; W—O*b*: 1.904 (5)—1.935 (5) Å; W—O*c*: 2.3185 (4)—2.3252 (5) Å; these bond lengths strictly follow the rule W—O*a* < W—O*b* < W—O*c*, which is in agreement with the Lindqvist-type polyoxidotungstate reported by Meng *et al.* (2006).

### Experimental

2,2'-bipyridine (0.5 mmoL 0.07 g) and *p*-carboxyphenylboronic acid were purchased from Jinan Henghua Science & Technology Co. Ltd. A mixture of 2,2'-bipyridine (0.5 mmol 0.07 g), tungstic acid (0.4 mmoL, 0.10 g), oxalic aicd (10 mmol, 0.09), *p*-carboxyphenylboronic acid (0.3 mmol, 0.05 g), and cobalt(II) sulfate heptahydrate (0.2 mmol, 0.05 g) in 14 ml distilled water was sealed in a 25 ml Teflon-lined stainless steel autoclave and was kept at 433 K for three days. Red crystals suitable for the X-ray experiment were obtained. Anal. Calc. for C_42_H_32_Co_2_N_8_O_23_W_6_: C, 22.53; H, 1.43; N, 5.01. Found: C, 22.26; H, 1.33; N, 4.85%.

### Refinement

All hydrogen atoms bound to carbon were refined using a riding model with distance C—H = 0.93 Å, *U*_iso_ = 1.2*U*_eq_(C). In the final difference Fourier map the highest peak is 2.60 Å from atom H1 and the deepest hole is 0.81 Å from atom W3. The highest peak is located in the voids of the crystal structure and may be associated with an additional water molecule. However, refinement of this position did not result in a reasonable model.

### Crystal data

**Table:**

[Co_2_(C_2_O_4_)(C_10_H_8_N_2_)_4_][W_6_O_19_]	*Z* = 1
*M**_r_* = 2237.72	*F*(000) = 1022
Triclinic, *P*1	*D*_x_ = 2.806 Mg m^−^^3^
Hall symbol: -P 1	Mo *K*α radiation, λ = 0.71073 Å
*a* = 9.4876 (15) Å	Cell parameters from 3530 reflections
*b* = 9.8548 (15) Å	θ = 2.5–27.3°
*c* = 14.174 (2) Å	µ = 13.67 mm^−^^1^
α = 90.769 (2)°	*T* = 293 K
β = 91.576 (2)°	Block, red
γ = 91.113 (2)°	0.12 × 0.10 × 0.08 mm
*V* = 1324.3 (4) Å^3^	

### Data collection

**Table:**

Bruker APEXII CCD diffractometer	4613 independent reflections
Radiation source: fine-focus sealed tube	3755 reflections with *I* > 2σ(*I*)
graphite	*R*_int_ = 0.027
φ and ω scans	θ_max_ = 25.0°, θ_min_ = 2.6°
Absorption correction: multi-scan (*SADABS*; Bruker, 2001)	*h* = −11→11
*T*_min_ = 0.291, *T*_max_ = 0.408	*k* = −11→11
9331 measured reflections	*l* = −16→16

### Refinement

**Table:**

Refinement on *F*^2^	Primary atom site location: structure-invariant direct methods
Least-squares matrix: full	Secondary atom site location: difference Fourier map
*R*[*F*^2^ > 2σ(*F*^2^)] = 0.031	Hydrogen site location: inferred from neighbouring sites
*wR*(*F*^2^) = 0.076	H-atom parameters constrained
*S* = 1.00	*w* = 1/[σ^2^(*F*_o_^2^) + (0.0375*P*)^2^ + 1.9139*P*] where *P* = (*F*_o_^2^ + 2*F*_c_^2^)/3
4613 reflections	(Δ/σ)_max_ = 0.001
368 parameters	Δρ_max_ = 2.41 e Å^−^^3^
0 restraints	Δρ_min_ = −1.10 e Å^−^^3^

### Special details

**Table:**

Geometry. All e.s.d.'s (except the e.s.d. in the dihedral angle between two l.s. planes) are estimated using the full covariance matrix. The cell e.s.d.'s are taken into account individually in the estimation of e.s.d.'s in distances, angles and torsion angles; correlations between e.s.d.'s in cell parameters are only used when they are defined by crystal symmetry. An approximate (isotropic) treatment of cell e.s.d.'s is used for estimating e.s.d.'s involving l.s. planes.
Refinement. Refinement of *F*^2^ against ALL reflections. The weighted *R*-factor *wR* and goodness of fit *S* are based on *F*^2^, conventional *R*-factors *R* are based on *F*, with *F* set to zero for negative *F*^2^. The threshold expression of *F*^2^ > σ(*F*^2^) is used only for calculating *R*-factors(gt) *etc*. and is not relevant to the choice of reflections for refinement. *R*-factors based on *F*^2^ are statistically about twice as large as those based on *F*, and *R*- factors based on ALL data will be even larger.

### Fractional atomic coordinates and isotropic or equivalent isotropic displacement parameters (Å^2^)

**Table:**

	*x*	*y*	*z*	*U*_iso_*/*U*_eq_	
C1	0.5864 (9)	0.5558 (9)	0.7481 (6)	0.039 (2)	
H1	0.6557	0.5423	0.7040	0.046*	
C2	0.4503 (10)	0.5210 (10)	0.7228 (7)	0.050 (3)	
H2	0.4275	0.4831	0.6638	0.060*	
C3	0.3498 (10)	0.5440 (10)	0.7869 (7)	0.050 (3)	
H3	0.2562	0.5220	0.7714	0.060*	
C4	0.3841 (9)	0.5996 (10)	0.8753 (6)	0.045 (2)	
H4	0.3150	0.6150	0.9192	0.054*	
C5	0.5239 (8)	0.6314 (8)	0.8957 (5)	0.0262 (18)	
C6	0.5713 (9)	0.6925 (8)	0.9874 (5)	0.031 (2)	
C7	0.4828 (10)	0.7231 (11)	1.0590 (7)	0.050 (3)	
H7	0.3869	0.7028	1.0521	0.061*	
C8	0.5343 (12)	0.7834 (12)	1.1405 (7)	0.062 (3)	
H8	0.4747	0.8067	1.1889	0.074*	
C9	0.6775 (12)	0.8085 (12)	1.1486 (7)	0.064 (3)	
H9	0.7157	0.8488	1.2035	0.077*	
C10	0.7625 (11)	0.7754 (10)	1.0781 (6)	0.046 (2)	
H10	0.8589	0.7931	1.0851	0.055*	
C11	0.8337 (10)	0.9655 (10)	0.8619 (7)	0.046 (2)	
H11	0.7816	0.9641	0.9166	0.055*	
C12	0.8660 (11)	1.0894 (10)	0.8234 (7)	0.052 (3)	
H12	0.8396	1.1705	0.8515	0.062*	
C13	0.9399 (12)	1.0868 (11)	0.7404 (8)	0.062 (3)	
H13	0.9621	1.1676	0.7105	0.074*	
C14	0.9796 (10)	0.9684 (10)	0.7032 (7)	0.047 (2)	
H14	1.0299	0.9669	0.6478	0.057*	
C15	0.9459 (8)	0.8496 (9)	0.7469 (6)	0.0306 (19)	
C16	0.9845 (8)	0.7135 (9)	0.7095 (6)	0.0294 (19)	
C17	1.0644 (9)	0.6960 (10)	0.6295 (6)	0.040 (2)	
H17	1.1000	0.7709	0.5981	0.048*	
C18	1.0897 (9)	0.5675 (10)	0.5977 (6)	0.043 (2)	
H18	1.1428	0.5541	0.5443	0.052*	
C19	1.0363 (9)	0.4584 (10)	0.6452 (6)	0.043 (2)	
H19	1.0522	0.3703	0.6243	0.051*	
C20	0.9589 (9)	0.4815 (9)	0.7240 (6)	0.035 (2)	
H20	0.9230	0.4076	0.7564	0.042*	
C21	1.0517 (8)	0.5600 (8)	1.0069 (5)	0.0255 (18)	
Co1	0.83213 (11)	0.65380 (11)	0.88211 (7)	0.0263 (3)	
N1	0.6258 (7)	0.6078 (7)	0.8323 (4)	0.0302 (16)	
N2	0.7116 (7)	0.7170 (7)	0.9974 (5)	0.0328 (17)	
N3	0.8729 (7)	0.8481 (7)	0.8254 (5)	0.0325 (16)	
N4	0.9334 (7)	0.6070 (7)	0.7558 (4)	0.0269 (15)	
O1	1.0227 (6)	0.6668 (6)	0.9616 (4)	0.0350 (14)	
O2	0.8466 (5)	0.4532 (6)	0.9359 (4)	0.0318 (13)	
O3	0.4860 (7)	0.8809 (7)	0.7694 (4)	0.0521 (18)	
O4	0.3221 (6)	1.0289 (6)	0.6381 (4)	0.0362 (14)	
O5	0.3335 (6)	1.1440 (6)	0.3892 (4)	0.0369 (15)	
O6	0.1293 (7)	1.1836 (7)	0.5300 (5)	0.0540 (18)	
O7	0.2326 (6)	0.9278 (6)	0.4737 (4)	0.0383 (15)	
O8	0.5000	1.0000	0.5000	0.0219 (16)	
O9	0.3985 (6)	0.7844 (6)	0.5844 (4)	0.0342 (14)	
O10	0.5765 (6)	0.7561 (6)	0.4464 (4)	0.0373 (14)	
O11	0.2952 (7)	0.6576 (7)	0.4139 (5)	0.0528 (18)	
O12	0.4107 (6)	0.8997 (6)	0.3357 (4)	0.0338 (14)	
W1	0.38156 (4)	0.80202 (4)	0.45023 (2)	0.03214 (12)	
W2	0.49167 (4)	0.92799 (4)	0.65534 (2)	0.03134 (12)	
W3	0.28645 (4)	1.10717 (4)	0.51619 (2)	0.03216 (12)	

### Atomic displacement parameters (Å^2^)

**Table:**

	*U*^11^	*U*^22^	*U*^33^	*U*^12^	*U*^13^	*U*^23^
C1	0.039 (5)	0.051 (6)	0.025 (4)	0.002 (5)	−0.004 (4)	−0.011 (4)
C2	0.047 (6)	0.057 (7)	0.044 (6)	0.004 (5)	−0.018 (5)	−0.008 (5)
C3	0.028 (5)	0.061 (7)	0.058 (6)	−0.010 (5)	−0.014 (5)	0.004 (5)
C4	0.031 (5)	0.063 (7)	0.041 (5)	−0.004 (5)	0.000 (4)	−0.002 (5)
C5	0.024 (4)	0.028 (5)	0.027 (4)	0.000 (4)	−0.003 (3)	0.001 (3)
C6	0.032 (5)	0.031 (5)	0.030 (4)	0.001 (4)	0.012 (4)	0.001 (4)
C7	0.038 (6)	0.064 (7)	0.049 (6)	−0.004 (5)	0.011 (5)	−0.014 (5)
C8	0.057 (7)	0.074 (8)	0.055 (7)	−0.002 (6)	0.027 (5)	−0.027 (6)
C9	0.072 (8)	0.083 (9)	0.036 (6)	−0.003 (7)	0.003 (5)	−0.030 (5)
C10	0.052 (6)	0.051 (6)	0.036 (5)	−0.007 (5)	0.003 (4)	−0.014 (5)
C11	0.044 (6)	0.039 (6)	0.056 (6)	0.007 (5)	0.010 (5)	−0.002 (5)
C12	0.064 (7)	0.023 (5)	0.067 (7)	0.006 (5)	−0.001 (6)	−0.008 (5)
C13	0.066 (8)	0.044 (7)	0.074 (8)	−0.007 (6)	−0.002 (6)	0.013 (6)
C14	0.053 (6)	0.042 (6)	0.047 (6)	−0.007 (5)	0.006 (5)	0.001 (5)
C15	0.021 (4)	0.038 (5)	0.033 (4)	−0.002 (4)	−0.006 (3)	0.001 (4)
C16	0.016 (4)	0.038 (5)	0.034 (4)	−0.009 (4)	−0.009 (3)	0.002 (4)
C17	0.039 (5)	0.049 (6)	0.031 (5)	−0.001 (5)	0.011 (4)	0.009 (4)
C18	0.036 (5)	0.056 (7)	0.038 (5)	0.002 (5)	0.009 (4)	−0.005 (5)
C19	0.047 (6)	0.044 (6)	0.037 (5)	0.004 (5)	0.003 (4)	−0.015 (4)
C20	0.034 (5)	0.028 (5)	0.042 (5)	0.000 (4)	0.002 (4)	−0.002 (4)
C21	0.016 (4)	0.029 (5)	0.032 (4)	−0.004 (3)	0.005 (3)	−0.005 (4)
Co1	0.0243 (6)	0.0289 (6)	0.0258 (6)	0.0001 (5)	0.0035 (4)	−0.0003 (5)
N1	0.026 (4)	0.034 (4)	0.031 (4)	0.001 (3)	0.005 (3)	−0.001 (3)
N2	0.034 (4)	0.031 (4)	0.033 (4)	−0.003 (3)	0.006 (3)	−0.011 (3)
N3	0.034 (4)	0.027 (4)	0.037 (4)	0.001 (3)	0.004 (3)	0.004 (3)
N4	0.026 (4)	0.028 (4)	0.027 (3)	−0.001 (3)	0.003 (3)	−0.002 (3)
O1	0.030 (3)	0.037 (4)	0.038 (3)	−0.002 (3)	−0.003 (3)	0.007 (3)
O2	0.023 (3)	0.036 (4)	0.035 (3)	−0.004 (3)	−0.005 (2)	0.004 (3)
O3	0.065 (5)	0.059 (5)	0.033 (3)	0.007 (4)	0.004 (3)	0.003 (3)
O4	0.032 (3)	0.048 (4)	0.029 (3)	0.001 (3)	0.014 (2)	−0.008 (3)
O5	0.037 (3)	0.041 (4)	0.032 (3)	0.009 (3)	0.000 (3)	0.000 (3)
O6	0.039 (4)	0.068 (5)	0.055 (4)	0.010 (4)	0.002 (3)	−0.014 (4)
O7	0.028 (3)	0.053 (4)	0.034 (3)	0.002 (3)	0.002 (3)	−0.008 (3)
O8	0.021 (4)	0.024 (4)	0.021 (4)	0.004 (3)	0.006 (3)	−0.003 (3)
O9	0.035 (3)	0.033 (4)	0.035 (3)	−0.004 (3)	0.008 (3)	0.000 (3)
O10	0.047 (4)	0.027 (3)	0.039 (3)	0.007 (3)	0.006 (3)	−0.006 (3)
O11	0.054 (4)	0.042 (4)	0.061 (4)	−0.016 (3)	0.009 (3)	−0.013 (3)
O12	0.033 (3)	0.046 (4)	0.022 (3)	0.002 (3)	0.003 (2)	−0.008 (3)
W1	0.0353 (2)	0.0295 (2)	0.0313 (2)	−0.00686 (16)	0.00560 (15)	−0.00922 (15)
W2	0.0381 (2)	0.0338 (2)	0.02229 (18)	0.00114 (16)	0.00478 (14)	−0.00123 (14)
W3	0.0274 (2)	0.0368 (2)	0.0325 (2)	0.00719 (16)	0.00465 (14)	−0.00600 (15)

### Geometric parameters (Å, °)

**Table:**

C1—N1	1.333 (10)	C19—C20	1.373 (11)
C1—C2	1.366 (13)	C19—H19	0.9300
C1—H1	0.9300	C20—N4	1.340 (10)
C2—C3	1.355 (13)	C20—H20	0.9300
C2—H2	0.9300	C21—O2^i^	1.254 (9)
C3—C4	1.388 (13)	C21—O1	1.271 (9)
C3—H3	0.9300	C21—C21^i^	1.528 (15)
C4—C5	1.379 (11)	Co1—O1	2.104 (6)
C4—H4	0.9300	Co1—N1	2.101 (7)
C5—N1	1.360 (9)	Co1—N4	2.105 (6)
C5—C6	1.479 (11)	Co1—N2	2.114 (6)
C6—N2	1.351 (10)	Co1—N3	2.119 (7)
C6—C7	1.370 (11)	Co1—O2	2.134 (6)
C7—C8	1.366 (13)	O2—C21^i^	1.254 (9)
C7—H7	0.9300	O3—W2	1.690 (6)
C8—C9	1.378 (15)	O4—W2	1.919 (6)
C8—H8	0.9300	O4—W3	1.926 (6)
C9—C10	1.343 (12)	O5—W3	1.904 (5)
C9—H9	0.9300	O5—W2^ii^	1.935 (5)
C10—N2	1.346 (10)	O6—W3	1.698 (6)
C10—H10	0.9300	O7—W3	1.915 (6)
C11—N3	1.325 (11)	O7—W1	1.931 (6)
C11—C12	1.377 (13)	O8—W3^ii^	2.3185 (4)
C11—H11	0.9300	O8—W3	2.3185 (4)
C12—C13	1.386 (14)	O8—W1	2.3240 (4)
C12—H12	0.9300	O8—W1^ii^	2.3240 (4)
C13—C14	1.339 (14)	O8—W2^ii^	2.3252 (5)
C13—H13	0.9300	O8—W2	2.3252 (5)
C14—C15	1.368 (12)	O9—W2	1.912 (6)
C14—H14	0.9300	O9—W1	1.915 (5)
C15—N3	1.327 (10)	O10—W1	1.914 (6)
C15—C16	1.491 (11)	O10—W3^ii^	1.914 (6)
C16—N4	1.336 (10)	O11—W1	1.696 (6)
C16—C17	1.391 (11)	O12—W1	1.922 (5)
C17—C18	1.367 (12)	O12—W2^ii^	1.920 (6)
C17—H17	0.9300	W2—O12^ii^	1.920 (6)
C18—C19	1.372 (12)	W2—O5^ii^	1.935 (5)
C18—H18	0.9300	W3—O10^ii^	1.914 (6)
			
N1—C1—C2	124.0 (8)	C1—N1—Co1	127.4 (5)
N1—C1—H1	118.0	C5—N1—Co1	114.6 (5)
C2—C1—H1	118.0	C10—N2—C6	118.9 (7)
C3—C2—C1	117.5 (9)	C10—N2—Co1	126.0 (6)
C3—C2—H2	121.3	C6—N2—Co1	115.1 (5)
C1—C2—H2	121.3	C11—N3—C15	118.4 (8)
C2—C3—C4	121.2 (9)	C11—N3—Co1	125.9 (6)
C2—C3—H3	119.4	C15—N3—Co1	115.7 (6)
C4—C3—H3	119.4	C20—N4—C16	119.2 (7)
C3—C4—C5	117.8 (8)	C20—N4—Co1	125.2 (6)
C3—C4—H4	121.1	C16—N4—Co1	115.4 (5)
C5—C4—H4	121.1	C21—O1—Co1	114.2 (5)
N1—C5—C4	121.5 (7)	C21^i^—O2—Co1	113.0 (5)
N1—C5—C6	116.4 (7)	W2—O4—W3	117.6 (2)
C4—C5—C6	122.1 (7)	W3—O5—W2^ii^	117.3 (3)
N2—C6—C7	120.6 (8)	W3—O7—W1	117.5 (3)
N2—C6—C5	115.4 (6)	W3^ii^—O8—W3	180.0
C7—C6—C5	124.0 (8)	W3^ii^—O8—W1	89.833 (16)
C8—C7—C6	120.4 (9)	W3—O8—W1	90.167 (16)
C8—C7—H7	119.8	W3^ii^—O8—W1^ii^	90.167 (16)
C6—C7—H7	119.8	W3—O8—W1^ii^	89.833 (16)
C7—C8—C9	117.8 (9)	W1—O8—W1^ii^	180.0
C7—C8—H8	121.1	W3^ii^—O8—W2^ii^	90.173 (13)
C9—C8—H8	121.1	W3—O8—W2^ii^	89.827 (13)
C10—C9—C8	120.7 (10)	W1—O8—W2^ii^	90.203 (14)
C10—C9—H9	119.7	W1^ii^—O8—W2^ii^	89.797 (14)
C8—C9—H9	119.7	W3^ii^—O8—W2	89.827 (13)
N2—C10—C9	121.6 (10)	W3—O8—W2	90.173 (13)
N2—C10—H10	119.2	W1—O8—W2	89.797 (14)
C9—C10—H10	119.2	W1^ii^—O8—W2	90.203 (14)
N3—C11—C12	123.5 (9)	W2^ii^—O8—W2	180.0
N3—C11—H11	118.2	W2—O9—W1	118.1 (3)
C12—C11—H11	118.2	W1—O10—W3^ii^	117.8 (3)
C13—C12—C11	116.4 (9)	W1—O12—W2^ii^	118.0 (3)
C13—C12—H12	121.8	O11—W1—O10	103.9 (3)
C11—C12—H12	121.8	O11—W1—O9	104.0 (3)
C14—C13—C12	120.2 (10)	O10—W1—O9	86.9 (2)
C14—C13—H13	119.9	O11—W1—O12	104.1 (3)
C12—C13—H13	119.9	O10—W1—O12	86.8 (2)
C13—C14—C15	119.8 (9)	O9—W1—O12	151.9 (2)
C13—C14—H14	120.1	O11—W1—O7	104.1 (3)
C15—C14—H14	120.1	O10—W1—O7	152.1 (2)
N3—C15—C14	121.6 (8)	O9—W1—O7	86.5 (2)
N3—C15—C16	115.2 (7)	O12—W1—O7	86.3 (2)
C14—C15—C16	123.2 (8)	O11—W1—O8	180.0 (3)
N4—C16—C17	121.1 (8)	O10—W1—O8	76.11 (17)
N4—C16—C15	115.8 (7)	O9—W1—O8	76.06 (17)
C17—C16—C15	123.0 (8)	O12—W1—O8	75.87 (17)
C18—C17—C16	119.2 (8)	O7—W1—O8	75.96 (18)
C18—C17—H17	120.4	O3—W2—O9	105.6 (3)
C16—C17—H17	120.4	O3—W2—O4	103.1 (3)
C17—C18—C19	119.5 (8)	O9—W2—O4	87.0 (2)
C17—C18—H18	120.2	O3—W2—O12^ii^	102.5 (3)
C19—C18—H18	120.2	O9—W2—O12^ii^	152.0 (2)
C18—C19—C20	118.9 (8)	O4—W2—O12^ii^	86.6 (2)
C18—C19—H19	120.6	O3—W2—O5^ii^	104.7 (3)
C20—C19—H19	120.6	O9—W2—O5^ii^	86.7 (2)
N4—C20—C19	122.1 (8)	O4—W2—O5^ii^	152.2 (2)
N4—C20—H20	118.9	O12^ii^—W2—O5^ii^	86.3 (2)
C19—C20—H20	118.9	O3—W2—O8	178.2 (2)
O2^i^—C21—O1	125.8 (7)	O9—W2—O8	76.08 (16)
O2^i^—C21—C21^i^	117.7 (9)	O4—W2—O8	76.11 (15)
O1—C21—C21^i^	116.4 (9)	O12^ii^—W2—O8	75.88 (15)
O1—Co1—N1	164.8 (2)	O5^ii^—W2—O8	76.08 (16)
O1—Co1—N4	93.3 (2)	O6—W3—O5	104.3 (3)
N1—Co1—N4	96.6 (2)	O6—W3—O7	103.2 (3)
O1—Co1—N2	92.9 (2)	O5—W3—O7	87.2 (2)
N1—Co1—N2	78.4 (3)	O6—W3—O10^ii^	104.2 (3)
N4—Co1—N2	172.1 (3)	O5—W3—O10^ii^	87.1 (2)
O1—Co1—N3	90.4 (2)	O7—W3—O10^ii^	152.6 (2)
N1—Co1—N3	103.0 (3)	O6—W3—O4	102.7 (3)
N4—Co1—N3	77.4 (3)	O5—W3—O4	153.0 (2)
N2—Co1—N3	97.7 (3)	O7—W3—O4	86.6 (2)
O1—Co1—O2	78.3 (2)	O10^ii^—W3—O4	86.3 (2)
N1—Co1—O2	89.4 (2)	O6—W3—O8	178.8 (2)
N4—Co1—O2	94.3 (2)	O5—W3—O8	76.81 (16)
N2—Co1—O2	91.7 (2)	O7—W3—O8	76.38 (16)
N3—Co1—O2	165.6 (2)	O10^ii^—W3—O8	76.26 (16)
C1—N1—C5	118.0 (7)	O4—W3—O8	76.16 (15)

Symmetry codes: (i) −*x*+2, −*y*+1, −*z*+2; (ii) −*x*+1, −*y*+2, −*z*+1.
